# Ancient Caucasian Wheats: A Contribution for Sustainable Diets and Food Diversity

**DOI:** 10.3390/foods11091209

**Published:** 2022-04-21

**Authors:** Francesca Nocente, Elena Galassi, Federica Taddei, Chiara Natale, Laura Gazza

**Affiliations:** CREA—Research Center for Engineering and Agro-Food Processing, Via Manziana 30, 00189 Rome, Italy; francesca.nocente@crea.gov.it (F.N.); elena.galassi@crea.gov.it (E.G.); federica.taddei@crea.gov.it (F.T.); chiara.natale@crea.gov.it (C.N.)

**Keywords:** *Triticum timopheevii*, *Triticum zhukovskyi*, food diversity, minor cereals, sustainable diets, ancient wheat

## Abstract

Through the centuries, the domestication and modern breeding of wheat led to a significant loss of genetic variation in the cultivated gene pool with a consequent decrease in food diversity. Current trends towards low-input and sustainable agriculture call for the revitalization and exploitation of ancient wheats, which represent a reservoir of biodiversity useful to ensure sustainable wheat production in the context of climate change and low-input farming systems. Ancient Caucasian wheat species, such as the hulled wheats *Triticum timopheevii* (tetraploid A^u^A^u^GG) and *Triticum zhukovskyi* (hexaploid A^u^A^u^A^m^A^m^GG), are still grown to a limited extent in the Caucasus for the production of traditional foods. These Caucasian wheats were grown in Italy and were analyzed for physical, nutritional and technological characteristics and compared to durum wheat. Both Caucasian species revealed a high protein content (on average 18.5%) associated with a low gluten index, mainly in *T. zhukovskyi*, and test weight values comparable to commercial wheats. The total antioxidant capacity was revealed to be the double of that in durum wheat, suggesting the use of ancient Caucasian wheats for the production of healthy foods. Finally, the technological and rheological results indicated that Caucasian wheats could be potential raw material for the formulation of flat breads, biscuits and pasta.

## 1. Introduction

Through the centuries, domestication and modern breeding made only three cereal species, rice, corn and wheat, provide almost 60% of the energy intake of the planet’s population [1]. The narrow focus of modern agriculture on intensive selection has led to a significantly reduced genetic diversity among wheat cultivars, since only few genotypes are cultivated on a large scale. The need for food diversification as well as the current demand for nutritionally healthy food products have driven a renewed interest in ancient wheats such as emmer, spelt and einkorn because of their desirable nutritional and putative health-beneficial traits [2,3]. Consequently, some neglected species and old varieties have been reintroduced in agriculture, having been recognized as interesting raw materials for the production of niche products. A superior quality, with reference to protein content, minerals and antioxidant compounds, along with minor adverse health effects in terms of allergy, intolerance and sensitivity, were observed in ancient wheats compared with the modern varieties [3,4,5,6,7,8,9]. Ancient Caucasian wheat species, such as the hulled wheats *Triticum timopheevii* (Zhuk.) Zhuk. subsp. *timopheevii* (tetraploid A^u^A^u^GG) and *Triticum zhukovskyi* Menabde et Erizian (hexaploid A^u^A^u^A^m^A^m^GG), investigated in the present study, have not been subjected to an extensive breeding activity, representing a reservoir of genes which could contribute to extending the biodiversity of cultivated wheats in order to better face climate fluctuations and biotic and abiotic stress. These two species were probably domesticated in Southern Turkey and Northern Syria and then transferred to Georgia, where they were cultivated as a mixture in a population called *Zanduri* which also comprises the diploid *Triticum monococcum* var. *hornemannii* (diploid A^m^A^m^) [10]. The genome analysis revealed that *T*. *zhukovskyi* originated from the hybridization of *T*. *timopheevii* with *T*. *monococcum* [11,12]. Wild *timopheevii* (Zhuk.) Zhuk. is also a primary genetic relative and gene donor to emmer wheat (*T. turgidum* subsp. *dicoccon* (Schrank) Thell.) and to common wheat (*T. aestivum* L.) [13,14]. It is worth noting that these ancient wheats are characterized by an immunity to the prevalent wheat diseases such as rusts, powdery mildew and *Fusarium* head blast, as well as a tolerance to salt and excessive humidity; additionally, they are well adapted to cool environments [13,15,16,17,18]. Their cultivation is currently limited to marginal areas for the production of traditional foods, particularly flat breads, and for feed, whereas straw is made into mats, carpets, baskets and is used for packing material [16].

To prevent the loss of Caucasian ancient wheat as an indispensable raw material for the preparation of typical foods and artifacts, a request for their inscription in the List of Intangible Cultural Heritage in Need of Urgent Safeguarding of UNESCO (United Nations Educational, Scientific, and Cultural Organization) was proposed in 2019 by the Minister of Environment, Protection and Agriculture of Georgia [19].

The reintroduction of the large-scale cultivation of undervalued cereal species, beyond showing acceptable agronomic performances, comes with the identification of feasible products (flours, breads, pasta, biscuits, beverages) appreciated by consumers and constituting a source of health-promoting bioactives.

Comparative studies on the grain quality of several ancient wheat species revealed a higher total phenolic and ferulic acids content in *T. thimopheevii* with respect to other ancient and common wheat varieties that were analyzed, along with a high antioxidant activity, balanced iron and zinc content and high protein content [16,20,21].

Considering the rising demand for ancient and undervalued crops in developed countries [22] and the paucity of scientific literature data about the nutritional and technological characteristics of these ancient species, the Caucasian wheats *T. thimopheevii* and *T. zhukovskyi*, grown in Italy, were analyzed in this work. The aim was to investigate both their capacity to be processed into foodstuff and their health-promoting potential, with a view to contributing to the sustainability, the resilience and the biodiversity of agrosystems and to fostering food diversification in the context of healthy and sustainable diets, pillars of the European ‘*Farm to Fork strategy*’ action plan [23].

## 2. Materials and Methods

### 2.1. Plant Material

**Figure 1:**
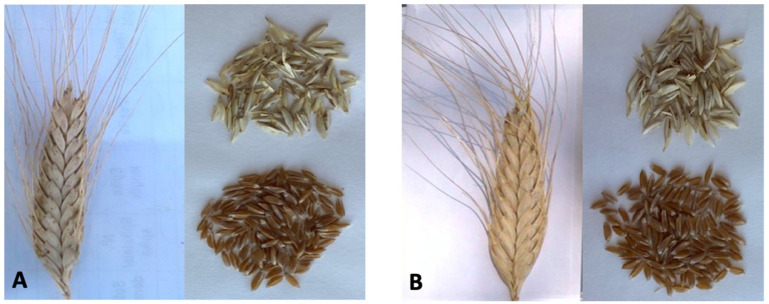
Ears, hulled and dehulled kernels of (**A**) *T. timopheevii* and (**B**) *T. zhukovskyi*.

*T. timopheevii* (accession Lonigo, Figure 1A) and *T. zhukovskyi* (accession Far 75, Figure 1B) were grown in 2020 in Montelibretti, Rome (Italy), at the experimental fields of the Research Center for Engineering and Agro-Food Processing (CREA-IT). The reference material was the *T. durum* cv San Carlo, largely used in Italy for pasta production. Each accession was grown in 10 m^2^ plots in randomized blocks with three replicates. The agronomic practices were those typical for durum wheat production in the selected area [24]. Immediately after harvest, the spikes from Caucasian wheats were threshed, and dehulled kernels were obtained by two subsequent steps using a bench micro-thresher (Marelli SpA, Milan, Italy); combined samples of grains from the three replicates were stored at 4 °C.

### 2.2. Grain Physical Analyses

The methods ISO 520:2010 [25] and ISO 7971-1:2009 [26] were used to determine the thousand kernel weight (TKW) and test weight (TW), respectively. The hardness index (HI) of the kernel was performed on 300 kernel samples by the Perten SKCS 4100 (Perten, Springfield, IL, USA), following the manufacturer’s operating procedure. The instrument was set at a range of hardness values between −40 and +120. The kernel length, width and thickness were recorded for 30 random kernels from each species using a calliper, and the average values were reported.

### 2.3. Chemical Characterization

All samples were milled to wholemeal flour using a laboratory mill (Cyclotec, FOSS, Hillerod, Denmark) at a 0.5 or 1.0 mm sieve, depending on the requirements of each analysis. All analyses were performed in triplicate. The sample moisture was measured using a thermobalance (Sartorius MA 40, Goettingen, Germany) at 120 °C just before the chemical analyses in order to express all data as dry weight (dw). Protein content was measured by micro-Kjeldhal nitrogen analysis according to the ICC 105/2 method [27], using as the conversion factor N × 5.7. The total and resistant starch (TS and RS) content was determined by enzymatic method using the Megazyme (Bray, Ireland) kits K-TSTA and K-RSTAR according to McCleary et al. [28] and McCleary et al. [29], respectively. The content of total dietary fiber (TDF) was measured using an enzymatic kit for fiber determination (Bioquant, Merck, Darmstadt, Germany) according to the AOAC Official Method 991.42 [30]. Protein, TS, RS and TDF content were expressed as percentage w/w. The total antioxidant capacity (TAC) was determined according to Ciccoritti et al. [31]. The total soluble phenolic content (TSPC) was determined using the Folin–Ciocalteau method as reported by Menga et al. [32], and the results were expressed as milligrams of ferulic acid equivalents per gram (mg FAE/g). Ash content was determined according to the approved method AACC 08-01.01 [33].

### 2.4. Rheological and Technological Tests

Semolina from durum and Caucasian wheats was obtained by Buhler MLU 202 mill (Utzwill, Switzerland). The total milling yield was considered as the percentage of the weight of semolina and flour fractions obtained from 100 g of kernels. The dry gluten content and gluten index were determined with the Glutomatic 2200 apparatus (Perten) according to the method ICC 158 [34]. Alveograph parameters (W, P and L) of semolina were obtained by Chopin Alveograph (Chopin, Villeneuve La Garenne, France) according to the manufacturer’s instructions. The SDS sedimentation test was assessed according to the standard method AACC 56-70.01 [33]. The AACC 56-81B method [33] was used for the determination of the falling number (FN) using the Perten 1500 system. Semolina color was evaluated by a Tristimulus colorimeter (ChromaMeter CR-400, Minolta, Milan, Italy) equipped with a D65 illuminant, using the CIELab color space coordinate b* (yellowness), a* (redness) and L* (lightness); brownness was expressed as 100-L*.

### 2.5. Statistical Analysis

Replicated results were expressed as mean ± standard deviation. A one-way analysis of variance was performed with MSTATC program (Michigan State University, East Lansing, MI, USA), followed by the Duncan multiple range test for a post-hoc comparison of means, applied to assess significant differences (*p* ≤ 0.05) for each considered parameter.

## 3. Results and Discussion

### 3.1. Physical Kernel Traits

Thousand kernel weight (TKW) and test weight (TW) are the main technological parameters indicating grain quality and play a large role in flour yield at milling [35]. The TKW values of de-hulled kernels were very similar in the Caucasian wheats, and they resulted in almost half of those of *T. durum* (Table 1). The TKW values were comparable to those obtained from the ancient wheats einkorn, spelt and emmer [36], but they were lower than those observed as the mean of more than 50 *T. timopheevii* accessions by Mikò et al. [37] and by Relina et al. [20], who found TKW values ranging from 33 to 39 g. These differences could be due to the agronomic practices, growing environment and genotypes used in the different studies. Similarly, no differences were observed between *T. timopheevii* and *T. zhukovskyi* for the TW values, which resulted in being statistically lower (*p* ≤ 0.05) than those observed in durum wheat (Table 1).

The kernel dimensions of Caucasian *Triticum* were significantly lower than those of durum wheat (Table 1), suggesting that the small kernel size of Caucasian wheats affected the kernel weight more than the TW, as already observed by Wang and Fu [38]. However, the TW value of 72 kg/hL, found in the two ancient wheats, met the current TW requirement for the No. 4 wheat class (TW ≥ 71 kg/hL) of Canada Western Amber Durum (CWAD) [39], whereas durum wheat cv San Carlo fell into the No. 1 CWAD class (TW ≥ 80 kg/hL) [39]. The mean values of 72 kg/hL of TW have also been reported for the ancient hulled wheats einkorn, spelt and emmer [40].

Endosperm texture in wheat exerts a strong indirect impact on a bulk of technological and rheological quality traits including flour yield, dough rheological properties, bread volume and crumb structure [41]. Almost all tetraploid cereal species are characterized by an extra-hard kernel texture with an SKCS hardness index (HI) > 80 [42], mainly due to the lack of expression of puroindolines proteins. Both *T. timopheevii* and *T. zhukovskyi* revealed a very hard kernel texture (HI > 80, Table 1), comparable to that of durum wheat. These results agree with Relina et al. [20] who classified the *T. timopheevii* kernels as hard-textured.

It is worth noting that even if the physical traits of Caucasian kernels showed significantly lower values than durum wheat (Table 1), their milling yield was satisfactory (61% and 70%, in *T. timopheevii* and *T. zhukovskyi*, respectively) and comparable to that of durum cv San Carlo (69%).

### 3.2. Chemical and Nutritional Traits

Besides their nutritional properties, proteins are important for the processing capacity of cereals, especially for the texture of poor-gluten quality foods. The whole wheat flour of *T. timopheevii* showed a significantly higher protein content (20.1%) than both *T. zhukovskyi* (16.9%) and durum wheat (14.3%) (Table 2).

A higher protein content in hulled ancient wheats with respect to modern wheat varieties was also observed in previous works [43,44,45], suggesting that the hulled wheat species have a better potential than modern wheat varieties for using nitrogen [43] and could therefore be considered as suitable crops for low input agriculture. However, one should take into consideration that the high protein content in ancient wheats is also ascribable to their low agronomic yield. As a consequence of the higher protein content, Caucasian wheats presented a lower total starch content than *T. durum* [46]. In any case, the very high protein and total starch content of about 62% make these wheats a valuable alternative raw material for producing highly nutritious cereal foods. The quantification of RS, i.e., the fraction of the starch that cannot be digested by human gastrointestinal enzymes, revealed, in all species, a RS content lower than the limit of 2% required for an adequate accuracy of the method used [29]. However, the method allowed for the discernment of a statistically different RS content between durum wheat (0.26%) and Caucasian wheats (0.17%). Dietary fiber is the main bioactive component of wheat grain, due to its health benefits in colon cancer prevention, prebiotic activity and modulation of blood glucose and insulin levels [47]. Durum wheat cv San Carlo had a significantly higher level of TDF when compared to both Caucasian wheats (Table 2). Generally, flours made from smaller kernels have a higher percentage of fiber; however, a lower content of dietary fiber in ancient wheat species has been reported in several studies related to the comparison between ancient and modern wheats [2,22,48]. Both *T. timopheevii* and *T. zhukovskyi* resulted in higher levels of minerals, as suggested by the significantly higher ash content (Table 2). A mean value of the ash content of 2% was also reported in spelt, einkorn and emmer [40,49]. The higher ash values in Caucasian wheats resulted from a higher share of outer kernel layers compared to durum wheat due to the smaller size of the grains. An adequate intake of minerals is an important contribution to human health, even if a higher content of minerals does not mean an improved uptake and bio-accessibility and kernels may also contain toxic metals [45].

Currently, antioxidant activity is the most common in vitro parameter that is used to assess or predict the potential benefits of phytochemical compounds. The level of TAC was significantly higher in *T. zhukovskyi* (+103%) and *T. timopheevii* (+98%) than in durum wheat cv San Carlo (Table 2). A higher antioxidant activity in *T. timopheevii* compared to durum wheat was also observed by Relina et al. [20]. The highest TAC level in ancient Caucasian wheats could not be ascribed to the presence of a major phenolics content compared to *T. durum*, since their TSPC was statistically lower than in the modern wheat cultivar (Table 2). Data on the phenolics content of ancient wheats usually [50] showed that wild tetraploid wheat ancestors had the lowest phenolic content, and, even if contradictory data are present in the literature, wild wheats do not seem to possess valuable characteristics for the improvement of TPC in wheat [48]. Hence, the very high level of TAC found in ancient Caucasian grains cannot be explained by their level of TSPC but rather by the occurrence of other bioactive compounds, such as carotenoids. This hypothesis should be confirmed by further studies; in any case, as *T. zhukovskyi* possesses the einkorn A^m^ genome, it can be assumed that it shares a high lutein content, einkorn being indicated as the wheat with the highest level of lutein [48,51]. Moreover, the higher yellow index (b*) shown by Caucasian wheats’ semolina, as reported in the following section, could reinforce this assumption.

### 3.3. Technological and Rheological Traits

Because of their poor-gluten quality, ancient wheats result in less structured doughs with a low elasticity and high extensibility [43]. The SDS-sedimentation test is one of the most useful single small-scale tests for screening for gluten strength and consequently for pasta-cooking and bread-making quality in durum wheat [52]. Significant differences (*p* ≤ 0.05) in SDS values (Table 3) were observed between the two ancient Caucasian wheats; in particular, *T. zhukovskyi* was considered as ‘poor gluten quality’, having an SDS value <30 mL, whereas ‘good gluten quality’ could be ascribed to *T. timopheevii*, which presented an SDS value in the range of 30–40 mL [53].

These results were in agreement with the gluten index values found in *T. zhukovskyi* and in *T. timopheevii* (Table 3). Indeed, according to the standard quality classes UNI 10709 [54] and UNI 10940 [55], *T. zhukovskyi* fell into the worst quality class, showing values slightly >1, whereas *T. timopheevii* showed a gluten index about three-fold lower than that recorded in durum wheat, falling into the quality class III. Despite the low gluten index, both Caucasian semolina showed a gluten content that was significantly higher than durum wheat (Table 3), due to the higher protein content (Table 2). It is worth noting that in *T. timopheevii* and *T*. *zhukovskyi*, the gluten content accounted for 85% and 90% of the total protein content, respectively, whereas in durum wheat cv San Carlo, it accounted for 73%.

Alveograph P and W values are indicators of dough elasticity and strength, respectively, and the L value is the indicator of dough extensibility. As expected, the poor quality of glutenin Caucasian wheat affected the rheological quality of semolina, as demonstrated by the W and P/L alveograph values (Table 3). The highest W value was observed in *T. durum* cv San Carlo, which met the requirements for the UNI 10709 [54] and UNI 10940 [55] standard quality class II, followed by *T. timopheevii* and *T. zhukovskyi*, which presented non-classifiable values (W < 100). The P/L ratio is a measurement of the balance between the elasticity and extensibility of dough and, with some exceptions, is higher than 1.0 in durum wheat [56], reflecting the tenacious and inextensible dough properties of this wheat species well. *T. timopheevii* showed a P/L value >1, similar to durum wheat cv San Carlo, whereas in *T. zhukovskyi* the low alveograph P value resulted in a significantly lower P/L ratio when compared to *T. timopheevii* and durum wheat. These results suggested that flours deriving from Caucasian wheats could be more suitable for being processed into pasta, flat breads and unleavened products than into traditional bread and baked products that require long leavening and processing.

The falling number (FN) is used to assess the baking quality of wheat flour in relation to the amylolytic enzymes activity, with which it is negatively correlated. FN values higher than 400 s were observed in the three analyzed species (Table 3), indicating a scarce amylolytic activity and, consequently, a poor bread-making performance in terms of crumb texture and low loaf volume. These data reinforced the idea, supported by alveograph tests, that Caucasian flours are optimal for being processed into pasta or flat breads, as already reported for einkorn, emmer and spelt wheat [57].

Kernel and milling products’ color is an important factor in anticipating the end-product color quality; it is used in the durum grain trade, and the higher the b* value, the more intense the yellow coloring of the sample. Elevated values of the b* parameter (Table 3) were found in Caucasian wheats’ semolina, mainly in *T. timopheevii,* which presented a higher b* value (+32%) than that of durum wheat semolina. On the contrary, the brown (100-L*) and red (a*) indexes, even if statistically different, were very similar in the three wheat species (Table 3).

## 4. Conclusions

The exploitation of ancient wheat species, besides playing a key role in plant breeding as a reservoir of useful genes, could contribute to providing new raw materials for the production of health-promoting foods, while increasing the agro-food biodiversity. The assessment of grain physical parameters, products’ feasibility, flours’ technological and rheological quality, and the presence of some health-promoting molecules revealed the ancient Caucasian wheats to be a valuable option for the entire supply chain, from farm to fork, meeting the main requirements that are used to evaluate the suitability of wheat for food production. Indeed, *T. timopheevii* and *T. zhukovskyi*, despite having a seed weight that was about half that of durum wheat, showed an excellent milling yield and an acceptable test weight, which suggests a promising use for processing. The technological and rheological parameters identified the Caucasian wheats as a potential raw material for the formulation of flat breads or biscuits, while the very high protein content could result in a good pasta-making capacity. Finally, the very high TAC level recorded in these wheats could satisfy the increasing demand for healthier and high-quality foods, encouraging the introduction of novel raw materials and products into diets, in developed countries as well. Future work will be necessary to evaluate the GxE effect on agronomical, nutritional and technological parameters and to investigate the most suitable technological processes and food.

## Figures and Tables

**Table 1:** Physical kernel traits of the two ancient Caucasian wheats and *T. durum* cv San Carlo.

	Thousand Kernel Weight (g)	Test Weight (kg/hL)	Hardness Index	Kernel Dimensions		
				Length (mm)	Width (mm)	Thickness (mm)
*T. timopheevii* accession Lonigo	28.0 ± 0.4 ^b^	72.2 ± 0.1 ^b^	83 ± 15 ^a^	8.7 ± 0.6 ^b^	2.3 ± 0.1 ^c^	2.4 ± 0.2 ^b^
*T.* z*hukovskyi* accession Far 75	27.7 ± 0.4 ^b^	72.0 ± 0.5 ^b^	85 ± 17 ^a^	8.9 ± 0.8 ^a^	2.7 ± 0.2 ^b^	2.5 ± 0.1 ^b^
*T. durum* cv San Carlo	56.2 ± 0.3 ^a^	84.3 ± 0.3 ^a^	84 ± 12 ^a^	8.1 ± 0.7 ^c^	3.7 ± 0.4 ^a^	3.5 ± 0.5 ^a^

Results are expressed as mean ± standard deviation for three replications. Within the same column, values with different letters indicate significant differences determined by Duncan’s test (*p* ≤ 0.05).

**Table 2:** Chemical and nutritional traits of the two ancient Caucasian wheats and *T. durum* cv San Carlo.

	Protein (%)	Total Starch (%)	TDF (%)	Ash (%)	TAC (mmol TEAC/kg)	TSPC (mg FAE/g)
*T. timopheevii* accession Lonigo	20.1 ± 0.8 ^a^	62.2 ± 0.19 ^b^	9.3 ± 0.2 ^c^	2.13 ± 0.01 ^a^	87.4 ± 0.5 ^b^	0.94 ± 0.05 ^b^
*T.* z*hukovskyi* accession Far 75	16.92 ± 0.03 ^b^	62.0 ± 0.3 ^b^	9.6 ± 0.2 ^b^	1.96 ± 0.02 ^b^	89.7 ± 0.3 ^a^	0.997 ± 0.005 ^b^
*T. durum* cv San Carlo	14.3 ± 0.5 ^c^	65.0 ± 0.8 ^a^	12.3 ± 0.3 ^a^	1.65 ± 0.01 ^c^	44.1 ± 0.3 ^c^	1.19 ± 0.04 ^a^

Results are reported as dry weight and expressed as mean ± standard deviation for three replications. Within the same column, values with different letters indicate significant differences determined by Duncan’s test (*p* ≤ 0.05). TDF = total dietary fiber; TAC = total antioxidant capacity; TEAC = trolox equivalent antioxidant capacity; TSPC = total soluble phenolic content; FAE = ferulic acid equivalents.

**Table 3:** Technological and rheological traits and semolina color of the two ancient Caucasian wheats and *T. durum* cv San Carlo.

	SDS Sedimentation Volume (mL)	Gluten Index (%)	Dry Gluten Content (%)	Alveograph Parameters		Falling Number (s)	Color		
				W	P/L		Yellow Index (b*)	Brown Index (100-L*)	Red Index (a*)
*T. timopheevii* accession Lonigo	34.5 ± 0.7 ^b^	34 ± 1 ^b^	17.13 ± 0.07 ^a^	29 ± 15 ^b^	1.2 ± 0.7 ^ab^	467 ± 1 ^b^	29.2 ± 0.2 ^a^	15.4 ± 0.2 ^a^	−2.69 ± 0.09 ^a^
*T.* z*hukovskyi* accession Far 75	22.5 ± 0.7 ^c^	1.3 ± 0.6 ^c^	15.3 ± 0.2 ^b^	9 ± 8 ^b^	0.8 ± 0.1 ^b^	476 ± 8 ^a^	27.7 ± 0.2 ^b^	15.6 ± 0.2 ^a^	−2.23 ± 0.09 ^b^
*T. durum* cv San Carlo	37.5 ± 0.7 ^a^	93 ± 1 ^a^	10.5 ± 0.1 ^c^	227 ± 21 ^a^	1.8 ± 0.1 ^a^	483 ± 2 ^a^	22.1 ± 0.2 ^c^	14.9 ± 0.5 ^b^	−2.3 ± 0.2 ^b^

Results are expressed as mean ± standard deviation for three replications. Within the same column, values with different letters indicate significant differences determined by Duncan’s test (*p* ≤ 0.05).

## Data Availability

The data presented in this study are available in this article.

## References

[1] Food and Agriculture Organization of the United Nation (FAO). https://www.fao.org/3/u8480e/u8480e07.htm.

[2] Arzani A., Ashraf M. (2017). Cultivated Ancient Wheats (*Triticum* spp.): A Potential Source of Health-Beneficial Food Products. Compr. Rev. Food Sci. Food Saf..

[3] Shewry P.R. (2018). Do ancient types of wheat have health benefits compared with modern bread wheat?. J. Cereal Sci..

[4] Dinu M., Whittaker A., Pagliai G., Benedettelli S., Sofi F. (2017). Ancient wheat species and human health: Biochemical and clinical implications. J. Nutr. Biochem..

[5] Brandolini A., Hidalgo A., Moscaritolo S. (2008). Chemical composition and pasting properties of einkorn (*Triticum monococcum* L. subsp. *monococcum*) whole meal flour. J. Cereal Sci..

[6] Shewry P.R., Hawkesford M.J., Piironen V., Lampi A.M., Gebruers K., Boros D., Andersson A.A.M., Aman P., Rakszegi M., Bedo Z. (2013). Natural Variation in Grain Composition of Wheat and Related Cereals. J. Agric. Food Chem..

[7] Molberg O., Uhlen A.K., Jensen T., Flaete N.S., Fleckenstein B., Arentz-Hansen H., Raki M., Lundin K.E., Sollid L.M. (2005). Mapping of gluten T-cell epitopes in the bread wheat ancestors: Implications for celiac disease. Gastroenterology.

[8] Spaenij-Dekking L., Kooy-Winkelaar Y., van Veelen P., Drijfhout J.W., Jonker H., van Soest L., Smulders M.J., Bosch D., Gilissen L.J., Koning F. (2005). Natural variation in toxicity of wheat: Potential for selection of nontoxic varieties for celiac disease patients. Gastroenterology.

[9] Picascia A., Camarca M., Malamisura R., Mandile M., Galatola M., Cielo D., Gazza L., Mamone G., Auricchio S., Troncone R. (2020). In celiac disease patients the in vivo challenge with the diploid *Triticum monococcum* elicits a reduced immune response compared to hexaploid wheat. Mol. Nutr. Food Res..

[10] Mori N., Kondo Y., Ishii T., Kawahara T., Valkoun J., Nakamura C. (2009). Genetic diversity and origin of timopheevi wheat inferred by chloroplast DNA fingerprinting. Breed. Sci..

[11] Dvořák J., Terlizzi P.D., Zhang H.B., Resta P. (1993). The evolution of polyploid wheats: Identification of the A genome donor species. Genome.

[12] Matsuoka Y. (2011). Evolution of Polyploid Triticum Wheats under Cultivation: The Role of Domestication, Natural Hybridization and Allopolyploid Speciation in their Diversification. Plant Cell Physiol..

[13] Zair W., Magos Brehm J. (2017). Triticum timopheevii. The IUCN Red List of Threatened Species.

[14] Vincent H., Wiersema J., Kell S., Fielder H., Dobbie S., Castaneda-Alvarez N.P., Guarino L., Eastwood R., Leόn B., Maxted N. (2013). A prioritized crop wild relative inventory to help underpin global food security. Biol. Conserv..

[15] Devi U., Grewal S., Yang C.Y., Hubbart-Edwards S., Scholefield D., Ashling S., Burridge A., King I.P., King J. (2019). Development and characterisation of interspecific hybrid lines with genome-wide introgressions from *Triticum timopheevii* in a hexaploid wheat background. BMC Plant Biol..

[16] Jorjadze M., Berishvili T., Shatberashvili E. (2014). The ancient wheats of Georgia and their traditional use in the southern part of the country. Emir. J. Food Agric..

[17] Brown-Guedira G.L., Gill B.S., Bockus W.W., Cox T.S., Hatchett J.H., Leath S., Peterson C.J., Thomas J.B., Zwer P.K. (1996). Evaluation of a collection of wild Timopheevii wheat for resistance to disease and arthropod pests. Plant Dis..

[18] Järve K., Jakobson I., Enno T. (2002). Tetraploid wheat species *Triticum timopheevii* and *Triticum militinae* in common wheat improvement. Acta Agron. Hung..

[19] Intagible Cultural Heritage UNESCO. https://ich.unesco.org/doc/src/47213-EN.doc.

[20] Relina L.I., Boguslavskyi R.L., Vecherska L.A., Didenko S.Y., Golik O.V., Sheliakina T.A., Pozdniakov V.V. (2018). Grain quality of tetraploid wheat *Triticum timopheevii* (zhuk.) zhuk. Plant Breed. Seed Prod..

[21] Engert N., Honermeier B. (2012). Characterization of grain quality and phenolic acids in ancient wheat species (*Triticum* sp). J. Appl. Bot. Food Qual..

[22] Zamaratskaia G., Gerhardt K., Wendin K. (2021). Biochemical characteristics and potential applications of ancient cereals-An underexploited opportunity for sustainable production and consumption. Trends Food Sci. Technol..

[23] The EU Farm to Fork Strategy for a Fair, Healthy and Environmentally-Friendly Food System. https://ec.europa.eu/food/system/files/2020-05/f2f_action-plan_2020_strategy-info_en.pdf.

[24] Quaranta F., Amoriello T., Aureli G., Belocchi A., D’Egidio M.G., Fornara M., Melloni S., Desiderio E. (2010). Grain yield, quality and deoxynivalenol (DON) contamination of durum wheat (*Triticum durum* Desf.): Results of national networks in organic and conventional cropping systems. Ital. J. Agric..

[25] International Organization for Standardization (ISO 2010) (2010). Cereals and Pulses-Determination of the Mass of 1000 Grains.

[26] International Organization for Standardization (ISO 2009) (2009). Determination of Bulk Density, Called Mass per Hectolitre-Part 1: Reference Method.

[27] International Association for Cereal Science and Technology (2003). ICC Standard Methods (Methods No. 105/2).

[28] McCleary B.V., Gibson T.S., Lugford D.C. (1997). Measurement of total starch in cereal products by amyloglucosidase-α-amylase method: Collaborative study. J. AOAC Int..

[29] McCleary B.V., McNally M., Rossiter P. (2002). Measurement of resistant starch by enzymatic digestion and selected plant materials: Collaborative study. J. AOAC Int..

[30] Cunniff P., Association of Official Analytical Chemists (1995). Official Methods of Analysis 991.

[31] Ciccoritti R., Taddei F., Nicoletti I., Gazza L., Corradini D., D’Egidio M.G., Martini D. (2017). Use of bran fractions and debranned kernels for the development of pasta with high nutritional and healthy potential. Food Chem..

[32] Menga V., Amato M., Phillips T.D., Angelino D., Morreale F., Fares C. (2017). Gluten-free pasta incorporating chia (*Salvia hispanica* L.) as thickening agent: An approach to naturally improve the nutritional profile and the in vitro carbohydrate digestibility. Food Chem..

[33] American Association of Cereal Chemists (2009). Approved Methods of Analysis.

[34] International Association for Cereal Science and Technology (1995). ICC Standard Methods (Methods No. 158).

[35] Dexter J.E., Marchylo B.A., Abeccassis J., Autran J.C., Feillet P. (2001). Recent Trends in Durum Wheat Milling and Pasta Processing: Impact on Durum Wheat Quality Requirements. Proceedings of the International Workshop on Durum Wheat, Semolina and Pasta Quality: Recent Achievements and New Trends.

[36] Belcar J., Sobczyk A., Sobolewska M., Stankowski S., Gorzelany J. (2020). Characteristics of Technological Properties of Grain and Flour from Ancient Varieties of Wheat (Einkorn, Emmer and Spelt). Acta Univ. Cibiniensis Ser. E Food Technol..

[37] Mikó P., Megyeri M., Molnár-Láng M., Kovács G. (2013). Characterization of *Triticum timopheevii* Zhuk. gene bank accessions for the development of synthetic amphiploid wheat lines. Acta Agron. Hung..

[38] Wang K., Fu B.X. (2020). Inter-relationships between test weight, thousand kernel weight, kernel size distribution and their effects on durum wheat milling, semolina composition and pasta processing quality. Foods.

[39] Canadian Grain Commission (2020). Wheat: Export Grade Determinants Tables for Canada Western Amber Durum (CWAD) Wheat. https://www.grainscanada.gc.ca/en/grain-quality/official-graingrading-guide/04-wheat/export-grade-determinants/cwad-en.html.

[40] Kulathunga J., Reuhs B.L., Zwinger S., Simsek S. (2021). Comparative study on kernel quality and chemical composition of ancient and modern wheat species: Einkorn, emmer, spelt and hard red spring wheat. Foods.

[41] Tsilo T.J., Hareland G.A., Chao S., Anderson J.A. (2011). Genetic mapping and QTL analysis of flour color and milling yield related traits using recombinant inbred lines in hard red spring wheat. Crop Sci..

[42] Gazza L., Conti S., Taddei F., Pogna N.E. (2006). Molecular characterization of puroindolines and their encoding genes in *Aegilops ventricosa*. Mol. Breed..

[43] Geisslitz S., Longin C.F.H., Scherf K.A., Koehler P. (2019). Comparative study on gluten protein composition of ancient (einkorn, emmer and spelt) and modern wheat species (durum and common wheat). Foods.

[44] De Santis M.A., Giuliani M.M., Giuzio L., De Vita P., Lovegrove A., Shewry P.R., Flagella Z. (2017). Differences in gluten protein composition between old and modern durum wheat genotypes in relation to 20th century breeding in Italy. Europ. J. Agric..

[45] Rachon L., Bobryk-Mamczarz A., Kiełtyka-Dadasiewicz A. (2020). Hulled Wheat Productivity and Quality in Modern Agriculture Against Conventional Wheat Species. Agriculture.

[46] Hucl P., Chibbar R.N. (1996). Variation for starch concentration in spring wheat and its repeatability relative to protein concentration. Cereal Chem..

[47] Prasadi V.P.N., Joye I.J. (2020). Dietary Fibre from Whole Grains and Their Benefits on Metabolic Health. Nutrients.

[48] Shewry P.R., Hey S. (2015). Do “ancient” wheat species differ from modern bread wheat in their contents of bioactive components?. J. Cereal Sci..

[49] Serban L.R., Păucean A., Man S.M., Chis M.S., Mureşan V. (2021). Ancient Wheat Species: Biochemical Profile and Impact on Sourdough Bread Characteristics—A Review. Processes.

[50] Laddomada B., Durante M., Mangini G., D’Amico L., Lenucci M.S., Simeone R., Piarulli L., Mita G., Blanco A. (2017). Genetic variation for phenolic acids concentration and composition in a tetraploid wheat (*Triticum turgidum* L.) collection. Genet. Resour. Crop Evol..

[51] Hidalgo A., Brandolini A., Pompei C., Piscozzi R. (2006). Carotenoids and tocols of einkorn wheat (*Triticum monococcum* ssp. monococcum L.). J. Cereal Sci..

[52] Peña R.J. (2000). Durum wheat for pasta and bread-making. Comparison of methods used in breeding to determine gluten quality-related parameters. Durum Wheat Improvement in the Mediterranean Region: New Challenges.

[53] AbuHammad W.A., Elias E.M., Manthey F.A., Alamri M.S., Mergoum M.A. (2012). Comparison of methods for assessing dough and gluten strength of durum wheat and their relationship to pasta cooking quality. Int. J. Food Sci. Technol..

[54] UNI Italian Organization for Standardization (1998). Durum Wheat Products for Pasta-Making—Definition, Characteristics and Quality Grades.

[55] UNI Italian Organization for Standardization (2001). Durum Wheat Products for Pasta-Making—Definition, Characteristics and Quality Grades.

[56] Quaglia G.B., Fabriani G., Lintas C. (1988). Other durum wheat products. Durum Wheat: Chemistry and Technology.

[57] Bobryk-Mamczarz A., Kiełtyka-Dadasiewicz A., Rachoń L. (2021). Usefulness of Hulled Wheats Grown in Polish Environment for Wholegrain Pasta-Making. Foods.

